# A Mini-Review of Pediatric Anthropometrics as Predictors of Future Insulin Resistance

**DOI:** 10.3389/fendo.2022.826430

**Published:** 2022-02-02

**Authors:** Sean DeLacey, Jami L. Josefson

**Affiliations:** ^1^ Department of Pediatrics, Division of Endocrinology, Ann & Robert H. Lurie Children’s Hospital of Chicago, Chicago, IL, United States; ^2^ Northwestern University Feinberg School of Medicine, Chicago, IL, United States

**Keywords:** anthropometrics, adiposity, obesity, insulin resistance, pediatrics

## Abstract

The impact of rising rates of childhood obesity is far reaching. Metabolic syndrome in children is increasing, yet for most children the consequences of excess adiposity will manifest in adulthood. Excess early fat accrual is a risk factor for future insulin resistance. However, certain types of fat and patterns of fat distribution are more relevant than others to metabolic risk. Therefore, adiposity measures are important. The link between childhood obesity and future insulin resistance was initially established with body mass index (BMI), but BMI is an in imperfect measure of adiposity. It is worthwhile to evaluate other anthropometrics as they may more accurately capture metabolic risk. While measures such as waist to height ratio are established as superior screening measures in adulthood - the findings are not as robust in pediatrics. Emerging evidence suggests that alternative anthropometrics may be slightly superior to BMI in identifying those youth most at risk of developing insulin resistance, but the clinical significance of that superiority appears limited. Increasing study is needed in longitudinal and varied cohorts to identify which pediatric anthropometric best predicts adult insulin resistance. We review alternative anthropometrics as predictors of future insulin resistance and identify current gaps in knowledge and potential future directions of inquiry.

## Introduction

Rising obesity rates have led to a commensurate unprecedented rise in associated comorbidities including insulin resistance. Insulin resistance is the body’s inability to effectively use insulin and a resultant increase in blood glucose levels. In most patients, excess fat is implicated in the development of insulin resistance. Insulin resistance is not just a potential precursor to diabetes mellitus, but is also a critical component of metabolic syndrome. Indeed, insulin resistance is often both a contributor to and a harbinger of other disturbances of metabolism including fatty liver disease and dyslipidemia (1, 2).

Obesity is defined as an accumulation of excess body fat to the extent that it may have an adverse effect on health (3). Obesity has become synonymous with definitions set by health organizations correlating with body mass index (BMI) thresholds. For example, in pediatrics, the World Health Organization (WHO) defines obesity as greater than two standard deviations above the Growth Reference median for BMI (4). Guidelines for screening and intervention are developed around these definitions. Therefore, it is critically important to assess the clinical impact of these definitions and measurements.

In contrast to obesity, adiposity is typically used as a general term to describe the degree of fat mass accumulation. While the typical population screening measure to define obesity is BMI, more technical/invasive measures such as body composition measurement by dual energy X-ray absorptiometry (DXA) are often used in research to measure adiposity. Indeed, the distribution and type of body fat, not simply the total body fat, has large implications for disease risk.

Anthropometrics are one method to estimate adiposity. Anthropometrics are broadly defined as physical measures of a person’s size and form that are physically obtained without the use of advanced equipment. While BMI is the most common anthropometric, other examples include waist circumference and skinfold thicknesses with calipers. Anthropometrics are of particular interest because of the ability to apply them to large populations without high associated costs or medical burden. The challenge is to identify anthropometrics best able to capture the type of adiposity that is predictive of disease.

Measuring adiposity is particularly challenging in children as their body composition and body metrics change physiologically (5). Inherently, proven measures in adults are not directly applicable to pediatric populations. Pubertal status may also change the utility of different anthropometrics because of the changing muscle and fat composition that occurs with sex hormone exposure (6, 7).

Work within the realm of obesity research has followed the progression of disease. While obesity rose in both adults and children in the early 1970s, the rates of obesity and metabolic complications are consistently higher in adults (8). Initial efforts aimed to identify the most clinically salient measures of adult adiposity. Multiple studies have indicated that waist circumference (WC) or waist to height ratio (WtHR) can better predict metabolic disease and insulin resistance than BMI alone in adults (9, 10). Given these findings and the rise of pediatric metabolic syndrome, subsequent efforts aimed to determine which measures of childhood adiposity best predict disease.

Childhood obesity is correlated with concurrent illness and childhood metabolic disease is an increasing phenomenon (11, 12). There is some evidence that alternative anthropometrics such as WtHR may have superiority to BMI z-score in predicting coincident disease such as non-alcoholic fatty liver disease (NAFLD) and insulin resistance (13, 14). However, there is no consensus around superior anthropometrics. Indeed a relatively recent metanalysis did not find significant evidence of superiority of WC or WtHR over BMI for insulin resistance related outcomes, though there was significant heterogenicity in results (11).

However, excess adiposity in childhood has more long-lasting and far-reaching impacts than current health status. Following the philosophy of the developmental origins of disease, insults or exposures in early life can independently influence later disease (15). Childhood obesity is a risk factor for adult insulin resistance even independent of adult obesity or childhood insulin resistance (16). Therefore, it is important to understand childhood adiposity not only as it predicts later adiposity and current insulin resistance, but how it influences future disease risk.

It is critical to be mindful and deliberate about how we measure childhood adiposity to best evaluate disease risk. In this review, we explore different measures of pediatric adiposity, specifically anthropometrics, that may predict insulin resistance. Specifically, we focus on the risk of future adult insulin resistance. We first establish the evidence linking childhood obesity as defined by BMI to adult insulin resistance and then compare different measures of adiposity as predictors. Finally, we identify gaps in the current literature and explore potential future avenues of study.

## BMI as a Predictor of Adult Insulin Resistance

When evaluating childhood adiposity as it predicts future insulin resistance, it is important to first discuss childhood BMI as a measurement. Given that BMI is the most common screening measure of adiposity, many studies have evaluated childhood BMI as a predictor of adult insulin resistance including several metanalyses. Indeed, in a comprehensive meta-analysis of seven cohorts, five cohorts found BMI it to be a statistically significant predictor of adult type 2 diabetes (17). In fact, the authors found diabetes to be the adult comorbidity most closely associated with childhood obesity. However, the associations and predictive value of BMI thresholds was relatively weak. For example, in children 7-11 years old, the odds ratio of diabetes per standard deviation increase of BMI was 1.78 (95% confidence interval of 1.51-2.10). However, the sensitivity of childhood BMI was poor for predicting adult diabetes and insulin resistance- at most, 40% of adults with diabetes would have met the threshold of excess adiposity (BMI>85%tile) in childhood (17, 18). A more recent cohort collaboration also found a significantly increased odds ratio of development of type 2 diabetes in adulthood as childhood BMI increased. Childhood BMI cut-points corresponding to 75th-90th percentile BMI based on CDC growth charts were determined to be at heightened risk for adult type 2 diabetes (19).

BMI has clear drawbacks as a measure of adiposity. BMI measures excess weight but does not differentiate between fat mass and non-fat mass. Additionally, it does not account for distribution of body fat and, in adults, central adiposity is more highly associated with adverse health outcomes than general adiposity (9, 20).

While the relationship between childhood BMI and adult insulin resistance is clearly of statistical significance, the weakness of its predictive value limits the utility of BMI as a screening measure. Therefore, it is worthwhile to explore different measures of adiposity to better capture risk. While the relationship between childhood BMI and adult-onset diabetes is relatively well studied, limited studies have evaluated different anthropometrics in childhood as they predict adult insulin resistance.

## Waist Circumference

The most studied BMI alternative is waist circumference (WC). As mentioned previously, it is established that adult WC correlates better with diabetes risk than BMI (10, 21). The stronger association likely exists because of the ability of WC to capture abdominal adiposity. However, the stronger relationship is not as well established in pediatrics.

The literature is particularly limited in evaluating childhood WC as a predictor of future insulin resistance. In general, evidence shows the association of childhood WC with future insulin resistance is stronger than that of BMI. However, the results are heterogenous with some studies showing lack of superiority (22, 23). The predictive ability of childhood WC for adult insulin resistance, as measured by sensitivity or area under the curve (AUC) in a receiver operating curve (ROC), remains low and is either similar to or only slightly superior to BMI (18, 23, 24). Therefore, similar to BMI, though a relationship between WC and adult insulin resistance clearly exists, it is difficult to identify thresholds of WC that reliably identify at risk youth.

## Sum of Skinfolds

Sum of skinfolds is a relatively frequently used alternative anthropometric to BMI although still rarely studied in longitudinal cohorts. Similar to WC, the literature suggests that childhood sum of skinfolds is either slightly better than or equivalent to BMI as a predictor of adult insulin resistance. For example, when defining childhood obesity by BMI thresholds alone investigators found no increased risk for adult diabetes, but the risk did exist when defining obesity by left subscapular skinfold (LSSF) thresholds (25). Similarly, a longitudinal study found the odds ratio of adult hyperglycemia higher in those with increased sum of skinfolds when compared to those with increased BMI in childhood (26). Finally, another study found that in a subsection of females, sum of skinfolds had a significantly higher association with fasting insulin levels in adulthood than both BMI and WC. While the study did not specifically evaluate the predictive ability of sum of skinfolds within this population for fasting insulin, it was found to have superior predictive ability for overall adult metabolic syndrome (22).

## Wrist Circumference

Several studies have shown a positive cross sectional relationship between wrist circumference and insulin resistance in children and adults (27, 28). However, the only study that has examined the relationship between pediatric wrist circumference and adult insulin resistance (as measured by euglycemic clamp) did not find that it predicted adult insulin resistance. Importantly, the study also did not find a relationship between childhood BMI and adult insulin resistance. The study was limited by a relatively small sample size and young adult population (29).

## Other Anthropometrics

Other anthropometric measurements include WC adjusted for height, weight adjusted for height, hip circumference, waist-hip-ratio, WtHR, conicity index, abdominal volume index, body adiposity index, and body shape index (**Table 1**). However, there is only one longitudinal study that examined all these measures as predictors of adult insulin resistance. Within the study, abdominal volume index performed the best among these indices at predicting insulin resistance in all three ways in which adult insulin resistance was measured. However, the overall predictive value was still relatively poor (AUC 0.610-0.615). While the predictive ability was superior to BMI in two of the three measures, it did not differ from other anthropometrics in a statistically significant way including: WC, WC adjusted for height, hip circumference, WtHR (23).

**Table 1:** Anthropometric measurement examples with their abbreviations and derived equations.

Anthropometric	Abbreviation	Calculation
Waist Circumference	WC	Not applicable
Wrist Circumference	WrC	Not applicable
Hip Circumference	HC	Not applicable
Waist-to-hip ratio	WHR	WC / HC
Waist-to-stature ratio Waist-to-height ratio	WSR WtHR	WC / height
Body adiposity index	BAI	(HC (cm) / height (m)) - 18
Body shape index	ABSI	WC (m) / (BMI (kg/m2) ^2/3^ x height (m) ^1/2^)
Body Roundness Index	BRI	364.2–365.5 x (1 − ((0.5 × WC (m) / π)^2^ / (0.5 × Height (m))^2^))^0.5^
Abdominal Volume Index	AVI	[2 cm (WC (cm)) ^2^ + 0.7 cm (WC (cm) − HC (cm))^2^] / 1,000
Conicity Index	CI	0.109^–1^ × WC (m) × (weight [kg] / height [m])^–1/2^
Sum of skinfold thickness	SSI	Triceps + subscapular (mm)

## Follow Up Period

One important note is that in all studies comparing the predictive ability of alternative anthropometrics, follow up is limited to young adulthood when insulin resistance or diabetes is not as prevalent as in later adulthood. It is reasonable to hypothesize the positive predictive value of anthropometrics may improve with longer follow up. Therefore, an extension of the current longitudinal what? is important to further elucidate the relationship.

## Ethnic and Racial Subclassification

Absent from most of the literature is a sub-group analysis by race/ethnicity. Previous evidence suggests racial/ethnic differences in thresholds of BMI/obesity at risk for insulin resistance (30, 31). For example, a UK study determined that threshold BMI for equivalent risk of concurrent insulin resistance was markedly lower in South Asian children than White European children (32). Additionally, some cross sectional studies suggest that different types of anthropometrics may better predict insulin resistance depending on the racial group, while others do not find a difference (33, 34).

The specificity of differing anthropometrics based on race is controversial. It is important to consider racial groups as social constructs. While an overlap exists between ancestry/ancestral genetics and race, they are not equivalent. Therefore, the differences in predictive abilities and predictive thresholds of anthropometrics between racial and ethnic subgroups need to be interpreted carefully both for their potential genetic and social etiologies. Many of the longitudinal cohort studies conducted have been in relatively homogenous and non-Hispanic White populations. Increased diversity of the study populations will improve generalizability and uncover potential differences in adiposity risk levels. Whether or not to use universal cut-off points for risk by anthropometrics or use ethnic and racial specific ones is a point of debate. Regardless, inclusion of diverse populations will ensure that risk thresholds are more broadly applicable.

## Reference Values

One of the difficult aspects of anthropometrics is the establishment of reference ranges and thresholds to define risk. As evidence accumulates around a particular type of adiposity measurement (i.e., BMI) the establishment of risk thresholds becomes clearer. When alternative measurements are not as widely used it is hard to extrapolate similar thresholds. Over time and in different populations, the thresholds will be different and thus it is important to recognize these limitations. Most of the longitudinal studies on prospective insulin resistance risk use thresholds based off each study’s sample data (i.e., the upper quartile of WC). Therefore, the findings are difficult to translate to direct clinical decisions. As data in the field grows larger, established population reference ranges may be possible.

## Addressing Puberty

The predictive value of adiposity can differ by sex and continuing to this difference is important (35–37). As previously mentioned, subtle anthropometric associations with future insulin resistance differ between females and males, but longitudinal cohorts have not completed sub-analysis by sex (22). The sex difference may be less pronounced in prepubertal children. Indeed, a subgroup analysis by pubertal status and age is important as adiposity changes across childhood, particularly in relation to sex hormone exposure. While both sexes increase their total fat stores during puberty, males gain relatively more fat free mass while females gain more fat mass (6). Regional body fat distribution also changes throughout puberty with males exhibiting increasing trunk and waist fat as they progress through puberty compared to females (7). While some of the cohort studies have performed subgroup analyses by pubertal status, most have not. Some evidence suggests there are differences in the predictive ability of anthropometrics by pubertal status, with one study finding that pre-pubertal anthropometrics are more strongly associated with adult diabetes than those in puberty (24). Continuing to explore these differences is crucial in evaluating anthropometrics as potential harbingers of future insulin resistance.

## Patterns in Adiposity Change

An increasing field of interest is the study of body composition and adiposity in the context of a trend or pattern of change. Multiple studies have evaluated the trajectory of obesity across childhood and into adulthood as a predictor of insulin resistance. For example, studies have already found those with a greater change in BMI during adolescence have increased future insulin resistance (38). Some have even begun evaluating trends of different adiposity measurements as they relate to short term outcomes (39, 40). As cohorts progress in age, additional studies of trajectories of other measures of adiposity, aside from BMI, on long term disease risk may reveal potential benefits to alternative measurements.

## Advanced Measurements of Adiposity

As previously discussed, while anthropometrics are one method to estimate adiposity, more advanced and accurate modes of measurement exist. Indeed, evidence exists that these measurements of adiposity better predict insulin resistance than anthropometrics. In a cross-sectional study of adult women, impaired glucose tolerance was better predicted by DXA-measured visceral adiposity than a variety of direct body measurements (41). Additionally, studies in adults and children have found body fat thresholds by air displacement plethysmography to be lower than those established by BMI or WC to define risk (42). In adolescents, visceral adiposity as measured by DXA was found to be associated with insulin resistance independent of BMI (43). To our knowledge no longitudinal studies exist evaluating the ability of more advanced measures of body fat measurement to predict future insulin resistance. Along with anthropometrics, this would be a valuable addition to the literature though will be limited in sample size.

## Limitations and Future Directions

In the limited existing research, there is consistent evidence that the association between some alternative adiposity measures such as WC or abdominal volume index, and future insulin resistance is slightly higher than for BMI. However, the predictive abilities of other measures remain low and suggests they may not have significant clinical superiority in identifying children at risk of developing insulin resistance. Given the stronger association and relatively stronger predictive power of alternative measures, the field of anthropometrics still deserves attention as a field of inquiry. There is reason to believe that as both diversity of cohorts and length of follow up increases, the differences in predictive ability between other anthropometrics and BMI may increase.

A large limitation to the field of knowledge is the need for large longitudinal cohort studies. While some anthropometrics have been available for some time, other measures of adiposity are relatively new and it will take time to determine their importance in screening as more data accumulates. As mentioned previously, the prevalence of insulin resistance increases with age and thus collecting data into early adulthood will not suffice to establish associations. Current longitudinal studies should be extended into later adulthood to allow for further exploration of the relationship of childhood adiposity and insulin resistance as its prevalence increases with age.

Additionally, the differences between other anthropometrics and BMI may be more significant as subgroup analysis is completed by sex, pubertal status, and racial/ethnic group. Among the few studies that exist on longitudinal risk of insulin resistance, few completed subgroup analysis by sex, pubertal stage or race/ethnicity. Given the evidence within cross sectional research for differences in adiposity between those of differing ancestries and sex hormone exposure, exploration into subgroup analysis should be completed in future research.

Finally, as the study of the adiposity rebound and adiposity trajectories progress, it will be worthwhile to trend alternative anthropometrics over time as well. The small differences in predictive power seen by static measurements may increase as they are studied in this manner.

While anthropometrics are important because of their widespread utility they are proxies for distinguishing different types of fat, for example subcutaneous and visceral fat. As more advanced non-invasive fat measurement methods such as air displacement plethysmography or ultrasound become less expensive and more accessible, the combination of these with anthropometrics in large cohorts will also help advance understanding of the impact of early fat accrual.

Overall, the use of alternative childhood anthropometrics to predict adult insulin resistance is relatively understudied and requires further development. As mentioned, the current literature is limited to a few studies. Most research comparing anthropometrics exists in cross-sectional cohorts where adiposity measures are correlated simultaneously to insulin resistance. However, the link between early adiposity and future insulin resistance is well established and deserves specific attention. We suggest that future prospective longitudinal studies should continue to incorporate anthropometrics in a variety of ways, particularly waist circumference and hip circumference as these are needed in the calculation of a variety of measurements.

## Author Contributions

SD and JLJ both conceived of the article content. SD prepared the first draft and both authors approved of the final version.

## Funding

SD’s work is supported by Ruth L. Kirschstein National Research Service Award T32 DK007169 from NIDDK. JLJ is supported by NIH R01DK118403.

## Conflict of Interest

The authors declare that the research was conducted in the absence of any commercial or financial relationships that could be construed as a potential conflict of interest.

## Publisher’s Note

All claims expressed in this article are solely those of the authors and do not necessarily represent those of their affiliated organizations, or those of the publisher, the editors and the reviewers. Any product that may be evaluated in this article, or claim that may be made by its manufacturer, is not guaranteed or endorsed by the publisher.
